# Increasing plasma ketamine concentrations decrease the minimum alveolar concentration of isoflurane in rabbits

**DOI:** 10.3389/fvets.2025.1604553

**Published:** 2025-06-19

**Authors:** Linda S. Barter, Bruno H. Pypendop

**Affiliations:** Department of Surgical and Radiological Sciences, School of Veterinary Medicine, University of California, Davis, Davis, CA, United States

**Keywords:** rabbit, anesthesia, ketamine, isoflurane, MAC

## Abstract

To evaluate the effects of increasing plasma ketamine concentration on isoflurane minimum alveolar concentration (MAC) in rabbits, six New Zealand white rabbits weighing 4.21 ± 0.35 kg were anesthetized with isoflurane in oxygen. Ketamine was given intravenously to target pseudo-steady-state plasma concentrations of 0.5, 1, 2, 4, 8, and 12 μg mL^−1^. MAC, heart rate, arterial blood pressure, body temperature, end-tidal carbon dioxide concentration, and plasma ketamine concentration were measured at each targeted ketamine concentration. A pharmacodynamic model was fitted to the plasma ketamine concentration-MAC data. The measured plasma ketamine concentrations were 0.53 ± 0.14, 1.25 ± 0.2, 2.64 ± 0.44, 5.11 ± 1.18, 8.96 ± 2.03, and 18.07 ± 4.2 μg mL^−1^, and isoflurane MAC values (% atm) were 1.66 ± 0.04, 1.39 ± 0.17, 1.16 ± 0.13, 1.02 ± 0.15, 0.86 ± 0.17, and 0.71 ± 0.06 for the six targeted plasma ketamine concentrations. MAC was significantly lower than baseline for the target concentration of 1 μg mL^−1^ and above. Heart rate was significantly reduced from baseline at plasma target ketamine concentrations of 2 μg mL^−1^ and higher. At target ketamine concentrations of 8 and 12 mcg mL^−1^, increased muscle tone and spontaneous movement were observed in some rabbits, requiring active cooling to maintain normothermia. Recoveries were unremarkable. MAC at plasma ketamine concentration C was predicted to be $1.85-\frac{1.25\times C}{2.96+C}$ . Increasing ketamine concentrations reduced isoflurane MAC in healthy female New Zealand White rabbits. Plasma ketamine concentrations between 1 and 4 μg mL^−1^ may elicit benefit with minimal adverse effects.

## 1 Introduction

General anesthesia in rabbits is associated with a higher risk of death than that reported in dogs and cats (1–3). Cardiovascular causes are among the most commonly attributed to anesthetic death. Surgical depth of anesthesia using inhalants in rabbits is associated with marked hypotension. At 1.5 MAC, isoflurane mean arterial pressures are reported as 40 ± 3 mm Hg (4). This hypotension has proven challenging to manage with exogenous catecholamines, with dopamine and phenylephrine administered at doses far higher than those used clinically in dogs and cats being minimally effective (5). Thus, investigating options for balanced anesthesia to reduce inhalant requirements and preserve cardiovascular performance may be especially beneficial in this species.

Ketamine is an anesthetic with analgesic properties, acting primarily at NMDA receptors, although activity at a range of other receptors has also been reported. Ketamine undergoes N-demethylation to produce norketamine, a non-competitive inhibitor of the NMDA receptor with reduced activity compared to the parent molecule (6, 7). Norketamine is further metabolized to hydroxynorketamine and dehydronorketamine, both of which appear to have minimal activity at NMDA receptors and are not considered to produce immobility in isolation (7). Although originally considered inactive, both hydroxynorketamine and dehydronorketamine are thought to play important roles in some therapeutic actions of ketamine, such as analgesia and antidepression, via activity at other central nervous system receptors (8, 9).

Ketamine given as a sole agent to dogs has been shown to increase heart rate, cardiac output, and arterial blood pressure (10). In conjunction with inhalants, ketamine has been shown to reduce MAC while preserving or improving cardiac output (11–13). As such, ketamine-inhalant combinations may be useful for general anesthesia in rabbits.

We hypothesized that ketamine would reduce isoflurane MAC in a plasma concentration-dependent manner while maintaining or improving blood pressure. The purpose of this study was to evaluate the effects of increasing plasma target concentrations on isoflurane MAC in rabbits and to collect data on physiologic variables at these MAC points.

## 2 Materials and methods

### 2.1 Animals

With the approval of the University of California, Davis, Institutional Animal Care and Use Committee (#20568), six adult female New Zealand white rabbits (*Oryctolagus cuniculus*) weighing 4.21 ± 0.35 kg were used in this study. Experiments complied with and were reported according to ARRIVE Guidelines. Rabbits were deemed healthy based on normal physical examinations performed at admission and again after a 2-week acclimation period. Rabbits were group housed in a rabbit-only room with a floor area measuring 3 × 4 m, bedded with shavings, and provided with plastic hutches. Rabbits were moved into individual cages measuring 74 × 72 × 47 cm (width × depth × height) for room cleaning and on study days. The rabbit room was on a 12-h light:dark cycle and maintained at a temperature of 21 ± 1°C. Animals were each provided 142 g per day of pelleted rabbit chow (High Fiber Rabbit Diet no. 5326, PMI Nutrition International, Brentwood, MO), Timothy hay, occasional green leafy vegetables, and free access to water. Housing conditions were within the requirements of the Animal Welfare Act and the Animal Welfare Regulations of the United States Department of Agriculture.^1^

### 2.2 Anesthesia and instrumentation

Anesthesia was induced by placing the rabbit into a transparent acrylic chamber into which 5% isoflurane (Isoflo; Abbott Animal Health, IL, United States) was delivered in 100% oxygen at 4 L min^−1^ via a Bain circuit. After loss of the righting reflex, anesthetic delivery was continued by a face mask attached to the Bain circuit with vaporizer at 4% and O_2_ flow reduced to 2 L min^−1^. Once at an appropriate anesthetic depth, as indicated by lack of withdrawal and palpebral reflexes, an orotracheal tube was placed (3.0–3.5 mm internal diameter) and connected to the Bain circuit with an oxygen flow of 200 mL kg^−1^ min^−1^ and vaporizer setting of 2%. Ophthalmic ointment (Puralube; Perrigo Company Plc, MN, United States) was placed in both eyes, and the animals were positioned in right lateral recumbency. Mechanical ventilation was instituted (Model 2000, Hallowell EMC, MA, United States) at a rate of 30 breaths min^−1^ and tidal volume adjusted to maintain end-tidal partial pressure of carbon dioxide (PE’CO_2_) from 30 to 35 mmHg.

A 22-gage 4.4 cm catheter (Introcan Safety, B. Braun Melsungen AG, Germany) was placed in the lateral saphenous vein for fluid and drug delivery. Lactated Ringer’s solution (Baxter Healthcare, Deerfield, IL, United States) was delivered intravenously (IV) throughout anesthesia at a rate of 3 mL kg^−1^ h^−1^ by a fluid pump (Heska Vet/IV 2.2, Heska Corporation, CO, United States).

A 20-gage 3.4 cm catheter (Safelet Cath; Exelint International Co., CA, United States) was placed into the auricular artery for direct blood pressure measurement and blood sampling.

Three sets of paired 25-gage hypodermic needles were placed subcutaneously on the ventral aspect of the shaved tail for use during MAC determination.

A multiparameter anesthesia monitor (Datex-Ohmeda S5; GE Healthcare Inc., IL, United States) was used to measure esophageal temperature, hemoglobin oxygen saturation via pulse oximetry, arterial pulse rate, blood pressure via a pressure transducer (DTX-plus; Argon Medical Devices, TX, United States), as well as end-tidal isoflurane concentration (FE’Iso) and PE’CO_2_. Prior to each anesthetic, the temperature probe and pressure transducer were calibrated against a certified mercury thermometer (Thermo Fisher Scientific Inc., MA, United States) and a mercury manometer, respectively. Additionally, the anesthetic gas analyzer was calibrated using four isoflurane secondary standards (0.514, 2.043, 2.381, and 3.332%), which spanned the range of measured isoflurane concentrations.

Normothermia (38.5–39.5°C) was maintained using an external heat pad (Hotdog; Augustine Medical Inc., MN, United States) and a forced air warming unit (Bair Hugger, 3 M Medical, Maplewood, MN, United States) as needed. If body temperature continued to rise despite removal of external heat, active cooling was initiated first by circulating room air, then by the application of a towel-wrapped bag of ice to the inguinal and axillary areas.

### 2.3 Experimental protocol

Isoflurane MAC and pharmacokinetics of ketamine during isoflurane anesthesia for this group of rabbits had been previously determined and published (14). In this study, isoflurane MAC was determined in duplicate at six different target plasma ketamine concentrations (0.5, 1, 2, 4, 8, and 12 μg mL^−1^). Each rabbit was anesthetized on two occasions at least 7 days apart. Three plasma ketamine concentrations were targeted on each day, initially selected at random but then arranged in ascending order for that day.

Ketamine (Zetamine; VetOne, ID, United States) was administered IV via the lateral saphenous catheter using a target-controlled infusion system (RUGLOOP I, Demed, Temse, Belgium) and syringe pump (PHD 2000 Programmable; Harvard Apparatus, MA, United States). The target-controlled infusion system loads the central compartment to the desired concentration rapidly and then adjusts the infusion rate every 10 s to maintain a pseudo-steady state plasma concentration, according to the equation *r* = *C_t_* × 0.581 × (0.138 + 0.530*e*^−0.176t^ + 0.148*e*^−0.016t^), where r is the infusion rate, *C_t_* is the target plasma concentration, and t is the infusion time in minutes.

Endotracheal tubes had been modified to contain a fine polypropylene catheter within their lumen, with the tip ending in the distal one-third of the endotracheal tube. This polypropylene catheter, connected via a 3-way stopcock, was used to collect end-tidal gas samples by hand into a glass syringe. The gas sample was then fed into the multiparameter monitor (Datex-Ohmeda S5; GE Healthcare Inc., IL, United States) for the measurement of isoflurane concentration and CO_2_ partial pressure by infrared analysis. Following any change in the vaporizer setting, end-tidal gas samples were collected by hand every 2–3 min until the desired FE’Iso was reached, then approximately every 5 min to confirm a stable FE’Iso. End-tidal gas samples were collected in triplicate, and the values were averaged for the final measurement.

Following at least 15 min of equilibration at a stable FE’Iso, end-tidal gas samples were collected and values for all monitored physiologic variables were recorded.

A supramaximal electrical stimulus (50 Hz, 6.5 ms, 15 V) (Grass Instruments, Quincy, MA, United States) was then delivered via one of the three pairs of needles for 1 min or until gross movement occurred. The needle pairs were used on a rotating basis to deliver successive stimuli.

If the rabbit moved in response to the stimulus, the FE’Iso was increased by 10%, and the procedure was repeated. Alternately, if the rabbit did not move, FE’Iso was decreased by 10%, and the procedure was repeated. The arithmetic mean of 2 successive concentrations that permitted and prevented movement was deemed the MAC. Minimum alveolar concentration was determined in duplicate, and the average calculated for each of the target plasma ketamine concentrations. Similarly, measured physiologic parameters were averaged across those values taken at FE’Iso used in MAC calculation for that target ketamine concentration.

Prior to starting ketamine administration and at each of the six MAC determinations (two each for three different ketamine concentrations) per study day, blood samples (0.75 mL each) were collected from the auricular arterial catheter. The total volume of blood removed from each animal per study day was approximately 2% of the total blood volume (15). Blood was immediately placed into tubes containing potassium ethylenediaminetetraacetic acid and centrifuged at 3,901 g and 4°C for 10 min. Plasma was then harvested and stored at −80°C until analyzed.

After each study, the arterial catheter, temperature probe, and stimulating needles were removed. Buprenorphine (0.03 mg kg^−1^; Reckitt Benckiser Pharmaceuticals Inc., VA, United States) was administered IV, and isoflurane administration was discontinued. Meloxicam (0.5 mg kg^−1^; Loxicom; Norbrook Laboratories Ltd., NC, United States) was administered subcutaneously following endotracheal tube removal. Once rabbits were ambulatory, the IV catheter was removed, and animals were placed in individual housing overnight to allow observation of behavior, appetite, and fecal output. They were returned to group housing the following day.

### 2.4 Plasma drug analysis

Plasma calibrators were prepared by dilution of the ketamine, norketamine, dehydronorketamine, and hydroxynorketamine working standard solutions (Cerilliant, Round Rock, TX, United States) with drug-free rabbit plasma to concentrations ranging from 0.05 to 12,000 ng mL^−1^. Quality control samples were included with each sample set. Prior to analysis, 0.2 mL of plasma was diluted with 100 μL of water containing d4-ketamine internal standard at 0.01 ng μL^−1^ (Cerilliant, Round Rock, TX). The samples were vortexed briefly to mix, and 4 mL of MTBE was added to each plasma sample. The samples were subsequently mixed by rotation (Glas-Col, Terre Haute, IN, United States) for 20 min at 40 revolutions per min, followed by centrifugation at 3,300 rpm (2,260 g) for 5 min at 4°C. The top organic layer was transferred to glass tubes, dried under nitrogen, dissolved in 140 μL of 5% acetonitrile (ACN) in water, with 0.2% formic acid, and 20 μL was injected into the liquid chromatography tandem mass spectrometry (LC–MS/MS) system.

Liquid chromatography tandem mass spectrometry in positive mode (LC–MS/MS(+)) was used to quantify the analytes using a TSQ Altis triple quadrupole mass spectrometer coupled with a Vanquish liquid chromatography system (Thermo Scientific, San Jose, CA). Chromatography used an Eclipse XDB-Phenyl 15 cm × 2.1 mm 5 μm column (Agilent, Palo Alto, CA, United States) and a linear gradient of ACN in water with a constant 0.2% formic acid at a flow rate of 0.4 mL min^−1^. The initial ACN concentration was held at 0% for 0.25 min, ramped to 50% over 6.6 min, and flushed for 0.2 min at 90% ACN, before re-equilibrating for 3.2 min at initial conditions.

Selective reaction monitoring (SRM) of initial precursor ion for ketamine (mass to charge ratio (*m/z*) 238.1), norketamine ((*m/z*) 224.1), dehydronorketamine ((*m/z*) 222.1), hydroxynorketamine ((*m/z*) 240.2), and the internal standard d4-ketamine ((*m/z*) 242.1) was used for detection and quantification. The response to the product ions for ketamine (*m/z*) 124.9, 206.9, norketamine (*m/z*) 125.1, 207.1, dehydronorketamine (*m/z*) 141.0, 142.0, hydroxynorketamine (*m/z*) 124.9, 151.1, and the internal standard d4-ketamine (*m/z*) 128.9 were plotted, and peaks at the proper retention time integrated, using Quanbrowser software (Thermo Fisher Scientific Inc. Waltham, MA, United States).

Precision and accuracy of the assay were determined by assaying quality control samples in replicates (*n* = 6/concentration) at 3 concentrations (0.15, 100, and 1,000 ng mL^−1^). Accuracy ranged from 97 to 108%, and precision was 3–14% for all analytes at all concentrations. The limit of quantitation and limit of detection, respectively, were 0.05 ng mL^−1^ and 0.01 ng mL^−1^ for ketamine, norketamine, and dehydronorketamine, and 0.1 ng mL^−1^ and 0.05 ng mL^−1^ for hydroxynorketamine.

### 2.5 Pharmacodynamic modeling

An inhibitory effect maximum model was fitted to the plasma ketamine concentration—MAC data using the following equation: $MAC_{C}=MAC_{0}-\frac{I_{\max}\times C}{IC_{50}+C}$ , where MAC_C_ is the isoflurane MAC at plasma ketamine concentration C, MAC_0_ is the baseline MAC, I_max_ is the maximum difference between MAC_0_ and the lowest MAC attained with ketamine, and IC_50_ is the plasma ketamine concentration at which 50% of I_max_ is produced. Non-linear mixed-effect modeling (population) methods were used in Phoenix NLME 8.5 (Certara, PA, United States). The first-order conditional estimation-extended least squares algorithm was used. Sigmoid and regular inhibitory maximum models and different error and covariance structures were attempted. The best-fitting model was selected based on the −2 log likelihood, inspection of the residual plots, and the precision of the parameters. The model estimated typical (population) values of the parameters and interindividual variability (random effects). Interindividual variability was omitted from the final model if the data were not sufficiently informative (shrinkage > 0.4).

### 2.6 Statistics

Power analysis based on a previous study in rabbits suggested that 6 animals would allow the detection of a 20% reduction in MAC with a power of 0.8 and an alpha level of 0.5 (16).

Pharmacodynamic parameters are presented as typical values (interindividual variability in %). All other data are presented as mean ± SD. The effect of target plasma ketamine concentration on isoflurane MAC was assessed by mixed model ANOVA with *post hoc* Sidak multiple comparisons. The effect of target plasma ketamine concentration on other measured values was analyzed by repeated measures ANOVA. If significant, post hoc t-tests were performed compared to baseline values, and sequentially rejective Bonferroni correction was used to adjust for multiple comparisons. The relationship between plasma ketamine, norketamine, hydroxynorketamine, and dehydronorketamine was examined by Pearson product–moment correlation. Significance was set at a *p*-value of < 0.05.

## 3 Results

**Figure 1:**
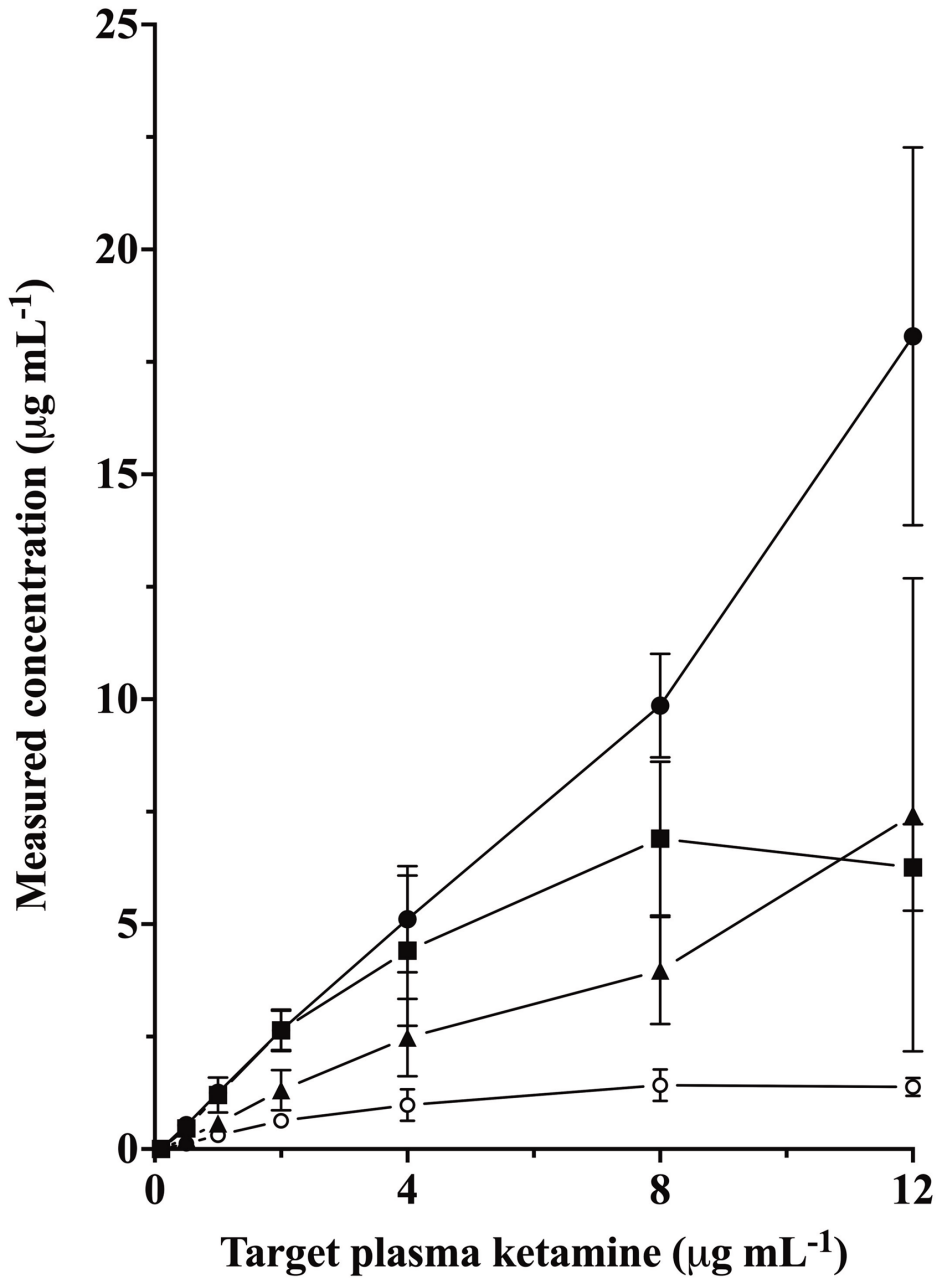
Measured plasma concentrations of ketamine (closed circle), norketamine (square), hydroxynorketamine (triangle), and dehydronorketamine (open circle) concentrations for the six targeted plasma ketamine concentrations of 0.5, 1, 2, 4, 8, and 12 μg mL^−1^ (mean ± SD) in six adult female isoflurane-anesthetized rabbits.

The MAC of isoflurane for rabbits in this study was 1.83 ± 0.12% atm (mean ± SD) (14). Measured plasma ketamine concentrations (mean ± SD) were 0.53 ± 0.14, 1.25 ± 0.2, 2.64 ± 0.44, 5.11 ± 1.18, 8.96 ± 2.03, and 18.07 ± 4.2 μg mL^−1^ for the target concentrations of 0.5, 1, 2, 4, 8, and 12 μg mL^−1^, respectively (Figure 1). Measured plasma norketamine, hydroxynorketamine, and dehydronorketamine are shown in Figure 1 and are highly correlated to measured plasma ketamine concentrations (*R* = 0.87, 1.00, 0.88, respectively).

**Figure 2:**
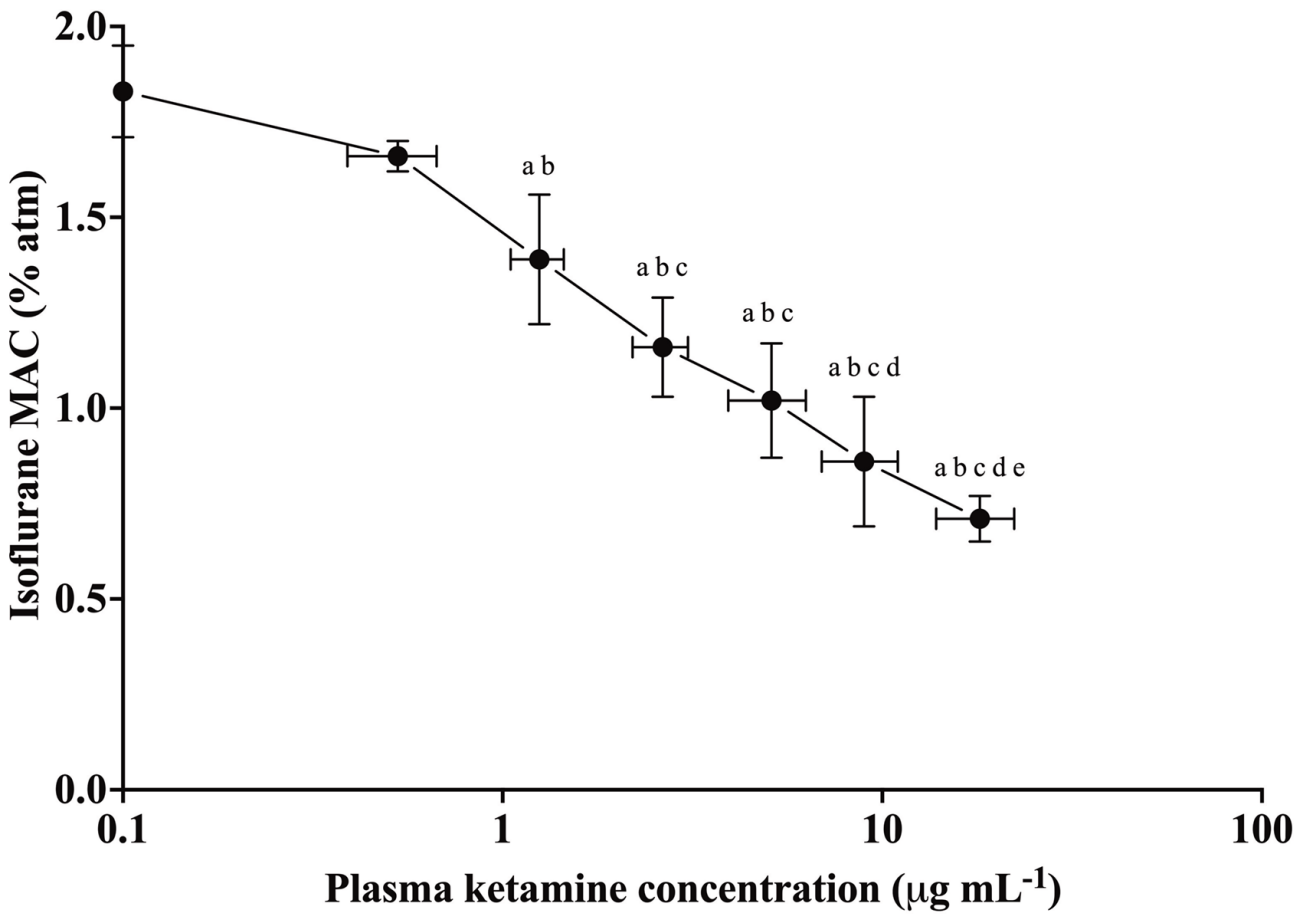
Minimum alveolar concentration of isoflurane (% atm) at baseline and six plasma ketamine concentrations (μg mL^−1^) for six adult female rabbits (mean ± SD). Significantly different from baseline (a), target ketamine concentrations (μg mL^−1^) 0.5 (b), 1 (c), 2 (d), and 4 (e).

**Figure 3:**
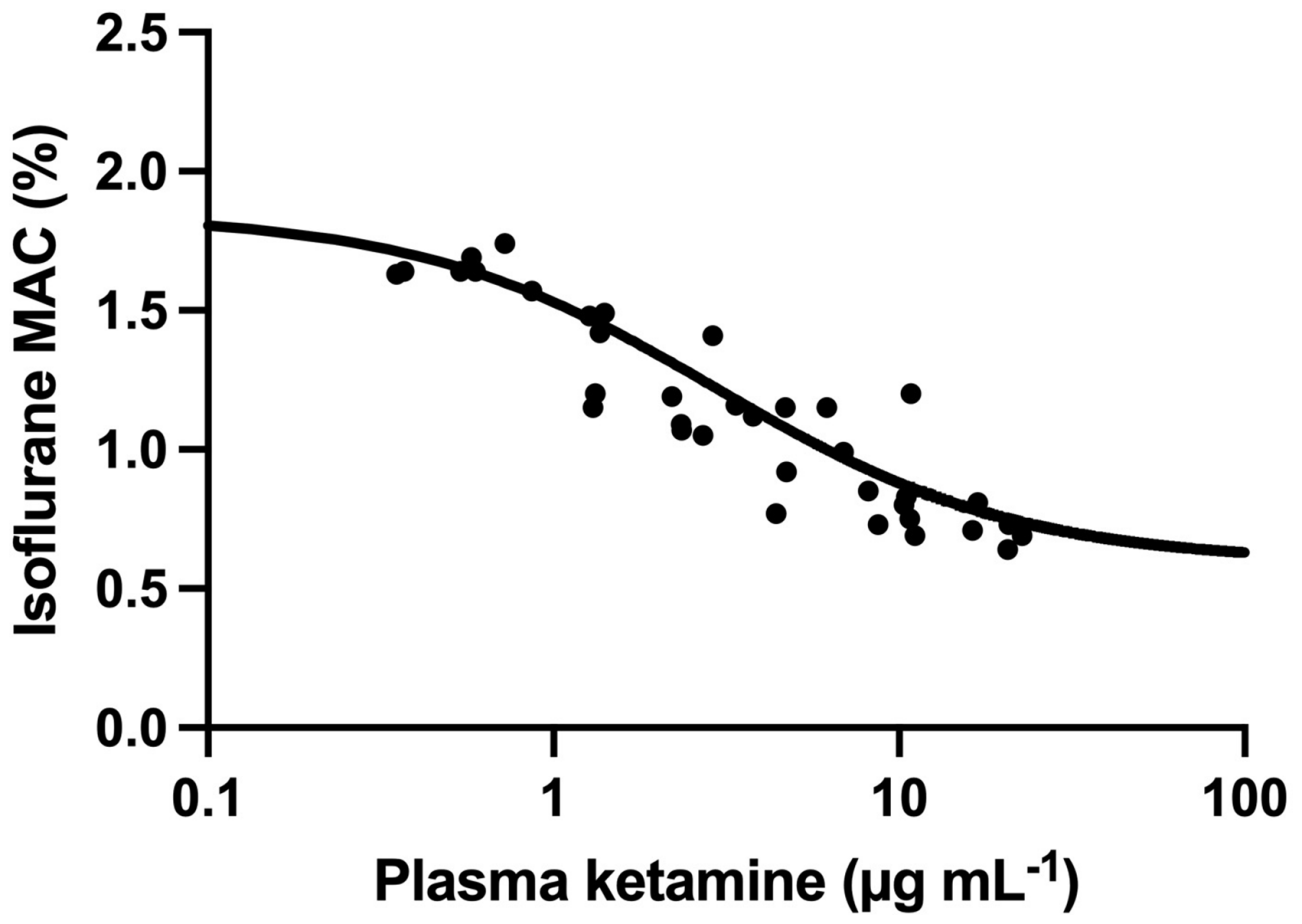
Observed (circle) and population predicted (line) isoflurane minimum alveolar concentration (MAC) at increasing plasma ketamine concentration. The predictions were obtained by fitting an inhibitory maximum effect model to the plasma ketamine concentration—MAC data.

**Figure 4:**
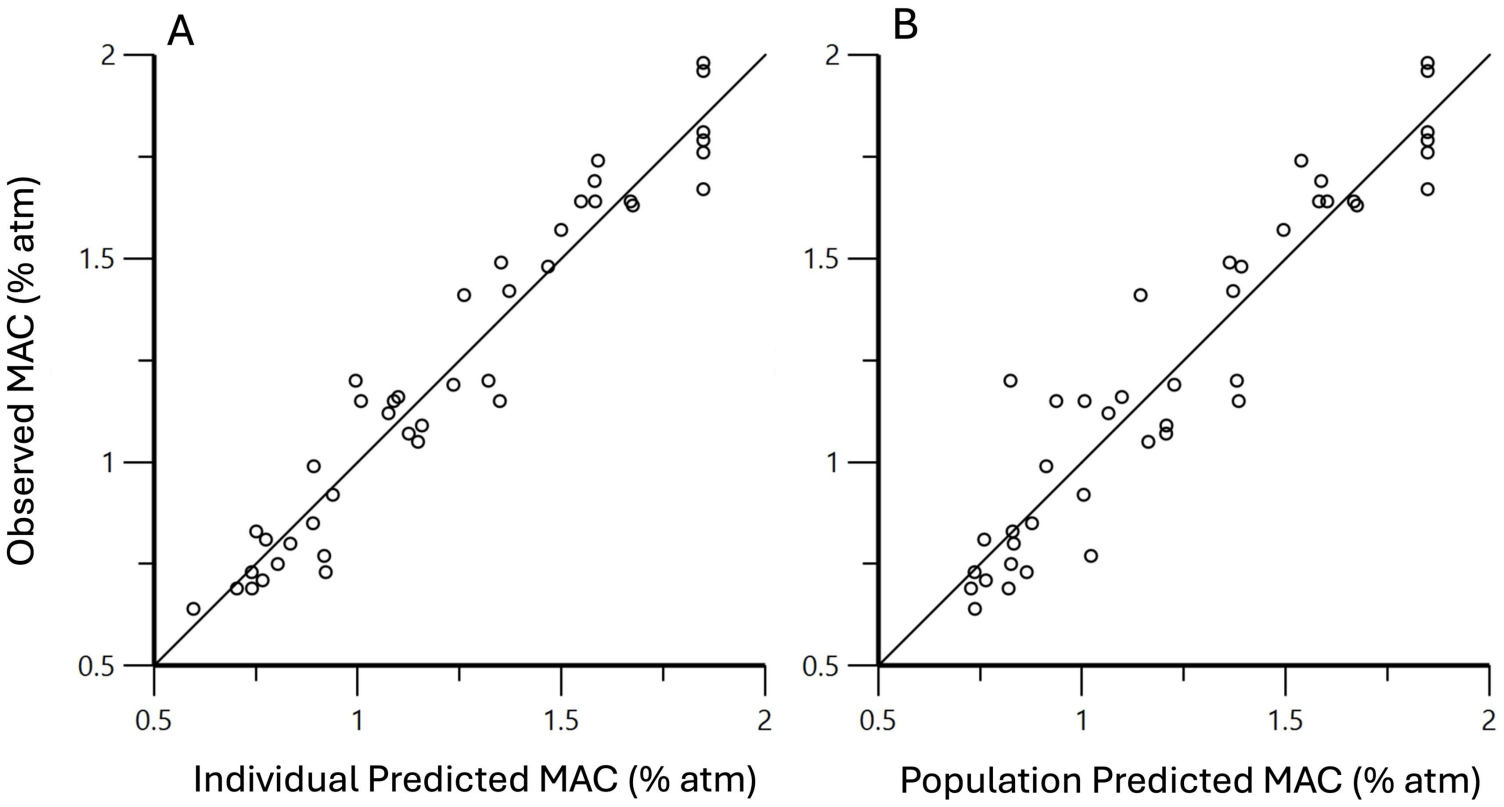
Using mixed effects modeling, the effect of ketamine on isoflurane MAC was predicted to be $MAC_{C}=1.85-\frac{1.25\times C}{2.96+C}$ , where C is the plasma ketamine concentration. Individual predicted MAC values versus observed MAC values are shown in **(A)**, and the population predicted MAC values versus observed MAC values in **(B)**. The solid line is the line of unity.

Isoflurane MAC values (% atm) were 1.66 ± 0.04, 1.39 ± 0.17, 1.16 ± 0.13, 1.02 ± 0.15, 0.86 ± 0.17, and 0.71 ± 0.06 for the target plasma concentrations of 0.5, 1, 2, 4, 8, and 12 μg mL^−1^, respectively (Figure 2). This corresponded to average MAC reductions of 9, 24, 36, 44, 53, and 61%, respectively, and was significant for the target concentration of 1 μg mL^−1^ and higher. MAC_0_, Imax (interindividual variability), and IC_50_ were predicted to be 1.85%, 1.25 (11) %, and 2.96 μg/mL, respectively. Based on those values, the effect of ketamine on the MAC of isoflurane was predicted to be $MAC_{C}=1.85-\frac{1.25\times C}{2.96+C}$ , where C is the plasma ketamine concentration (Figure 3). To evaluate the performance of this model, individual predictions and population predictions for MAC versus observed MAC values are shown in Figures 4A,B, respectively.

Values for heart rate, systolic, mean, and diastolic arterial pressure, PE’CO_2_, and body temperature at baseline and the six targeted plasma ketamine concentrations at the time of MAC determination are presented in Table 1. Heart rate was significantly lower than baseline values for plasma target ketamine concentrations of 2, 4, 8, and 12 μg mL^−1^; however, there were no differences in any other measured values. All six rabbits had heat support removed during all anesthetics as targeted plasma ketamine concentrations increased. Active cooling was needed to maintain body temperature within normal limits for 2/6 and 5/6 rabbits at target concentrations of 8 and 12 μg mL^−1^, respectively. Accompanying the increase in body temperature at the highest targeted plasma ketamine concentrations was increased muscle tone, rigidity, and spontaneous movements (twitching, paddling). Affected rabbits were those that measured plasma ketamine concentrations above the targeted value.

**Table 1:** Physiological parameters measured in six rabbits anesthetized at isoflurane minimum alveolar concentrations in the absence of, and at six targeted plasma concentrations of, ketamine.

Target [ketamine] (μg mL^−1^)	0	0.5	1	2	4	8	12
HR (bpm)	239 ± 16	224 ± 23	222 ± 26	210 ± 15^†^	204 ± 11^†^	193 ± 15^†^	183 ± 17^†^
SAP (mm Hg)	67 ± 10	63 ± 4	65 ± 11	67 ± 14	62 ± 13	65 ± 14	61 ± 8
MAP (mm Hg)	49 ± 6	44 ± 4	46 ± 5	49 ± 8	47 ± 8	49 ± 6	47 ± 7
DAP (mm Hg)	41 ± 6	37 ± 3	38 ± 4	42 ± 6	40 ± 7	43 ± 5	41 ± 6
PE’CO_2_ (mm Hg)	31 ± 2	31 ± 2	32 ± 2	31 ± 3	31 ± 1	32 ± 1	31 ± 1
Temp (°C)	38.7 ± 0.1	38.9 ± 0.3	38.9 ± 0.2	39.0 ± 0.4	39.0 ± 0.3	39.1 ± 0.4	39.1 ± 0.3

Mean ± SD heart rate (HR), systolic (SAP) mean (MAP), and diastolic (DAP) arterial pressures, end-tidal carbon dioxide (PE’CO_2_), and esophageal body temperature (Temp). † significantly different from baseline.

All animals recovered uneventfully without the need for sedation, additional analgesics, or supplemental care.

## 4 Discussion

In this study, isoflurane MAC was decreased in a plasma concentration-dependent manner by the six plasma ketamine concentrations evaluated. The maximal MAC reduction achieved in this study averaged 61% at the highest target plasma concentration of 12 μg mL^−1^, which is less than that reported in some other studies of dogs and cats. Isoflurane MAC was decreased by 70–92% at a target plasma ketamine concentration of 8 μg mL^−1^ in dogs (13). Measured plasma ketamine concentrations in that study were extremely variable, and at the 8 μg mL^−1^ target, actual concentrations were 13.03 ± 10.49 μg mL^−1^. Ketamine has been shown previously to reduce isoflurane MAC in rabbits by 24% following the administration of a ketamine loading dose of 1 mg kg^−1^, followed by a constant rate infusion of 40 μg kg^−1^ min^−1^ (17). That study only investigated a single ketamine dosage, and plasma ketamine concentrations were not measured.

The performance of our pharmacokinetic model and pseudo-steady state delivery system was reasonable at most target concentrations. Mean measured concentrations were greater than the target for all 6 targets, with the ratio of mean actual to target concentration of 105, 125, 132, 128, 123, and 151%, respectively. The actual plasma ketamine concentrations for the highest and lowest targets are outliers. Our pharmacokinetic model was created after the administration of a moderate dose (5 mg kg^−1^) of ketamine at an isoflurane concentration of 0.75 MAC (14). It is possible that this model, derived from declining plasma concentrations following a moderate dose, was less able to predict the disposition of ketamine when high plasma concentrations were targeted. Additionally, ketamine may influence its disposition as increasing concentrations of ketamine coupled with decreasing isoflurane concentrations alter hemodynamics and hepatic blood flow.

Despite a decrease in isoflurane MAC, there were no significant differences in arterial blood pressure measurements across the six treatments compared to baseline. Although the lack of significance cannot be interpreted as a lack of difference, the data themselves show no meaningful improvement in arterial blood pressure with increasing plasma ketamine concentrations and decreasing isoflurane concentrations. This may suggest the limited usefulness of ketamine as an anesthetic adjunct to inhalant anesthetics if the goal is to improve inhalant-induced hypotension. Rabbits in this study were hypotensive despite being anesthetized at approximately 1 MAC. Since the effective inhalant concentration for surgery is 30–40% higher than MAC (18), it is likely that the severity of the hypotension would be greater at such clinically relevant doses. Further studies are needed to determine if the use of ketamine as an anesthetic adjunct may hold cardiovascular benefits at such doses.

Interestingly, the heart rate was significantly lower than baseline at target ketamine concentrations of 2 μg mL^−1^ and above. Ketamine, as a sympathetic stimulant, is typically associated with increasing heart rates. In dogs anesthetized with increasing plasma ketamine concentrations and isoflurane 1.25 MAC, heart rate increased by approximately 30% over baseline values with target ketamine concentrations of 2 μg mL^−1^ and above (11). Isoflurane-anesthetized rabbits have been reported to experience profound systemic vasodilation (4). In this study, arterial blood pressure remained unchanged despite the decreasing heart rate. Consequently, either stroke volume or systemic vascular resistance must have increased. Ketamine infusion with concurrent decrease in isoflurane concentration has been shown to increase systemic vascular resistance in isoflurane-anesthetized dogs (11). Unfortunately, in the absence of more advanced cardiovascular monitoring, we can only speculate as to the underlying cardiovascular effects of ketamine in isoflurane-anesthetized rabbits in this study.

Rabbits were mechanically ventilated during this study to increase the ease of obtaining end-tidal gas samples for isoflurane concentration measurement. As such, PaCO_2_ was controlled by the investigators, so the respiratory impact of increasing ketamine and decreasing isoflurane as an anesthetic management technique cannot be assessed from the data obtained.

The increase in body temperature and muscle tone seen at the highest ketamine target concentration of 12 μg mL^−1^ limits the clinical usefulness of infusions producing such plasma concentrations for inhalant dose reduction in rabbits. This effect is consistent with previous reports of dogs and cats (11, 12). With the administration of increasing doses of ketamine to isoflurane-anesthetized dogs, core body temperature increased in a dose-dependent manner from 37.2 ± 0.6°C at 1.25MAC isoflurane to 38.9 ± 0.5°C at a plasma ketamine concentration of 24.16 μg mL^−1^ in the face of active cooling (11). In isoflurane anesthetized cats, ketamine infusions of 23, 46, and 115 μg kg^−1^ min^−1^ produced significant increases in core body temperature from a baseline of 38.2 ± 0.2 to 38.7 ± 0.4, 39.0 ± 0.2, and 38.9 ± 0.3°C, respectively (12). Both studies reported spontaneous movement at higher ketamine doses but provided no further details of the number of animals or specific plasma concentrations affected.

Although recoveries were uncomplicated in this study, prolonged recoveries following ketamine infusions in isoflurane anesthetized cats have been reported (12). In the aforementioned cat study, norketamine was implicated as a contributing factor to long recovery times, although it was not measured in that study. Plasma concentrations of ketamine metabolites increased as plasma ketamine concentrations increased in the present study, but we did not look at the effect of prolonged infusion times on metabolite concentrations. This would be deserving of further investigation prior to the clinical use of ketamine infusions during longer anesthetics.

This study investigated the effect of ketamine during isoflurane anesthesia in a small number of healthy female rabbits of a single breed, and results may differ in a clinical setting with sick animals.

Ketamine significantly reduced isoflurane MAC in rabbits without a significant effect on mean arterial blood pressure. Higher dosages were associated with increased muscle activity and heat production, requiring active cooling, making them undesirable for clinical use. Plasma ketamine concentrations in the range of 1–4 μg kg^−1^ may provide benefit in reducing arterial blood pressure during inhalant anesthesia in rabbits.

## Data Availability

The raw data supporting the conclusions of this article will be made available by the authors, without undue reservation.

## Footnotes

^1^https://www.aphis.usda.gov/sites/default/files/AC_BlueBook_AWA_508_comp_version.pdf, accessed March 30, 2025.
