# Supplementary figures and images for: Increasing plasma ketamine concentrations decrease the minimum alveolar concentration of isoflurane in rabbits
Source: Front Vet Sci. 2025 Jun 19;12:1604553. doi: 10.3389/fvets.2025.1604553 (PMC12221929; doi:10.3389/fvets.2025.1604553)

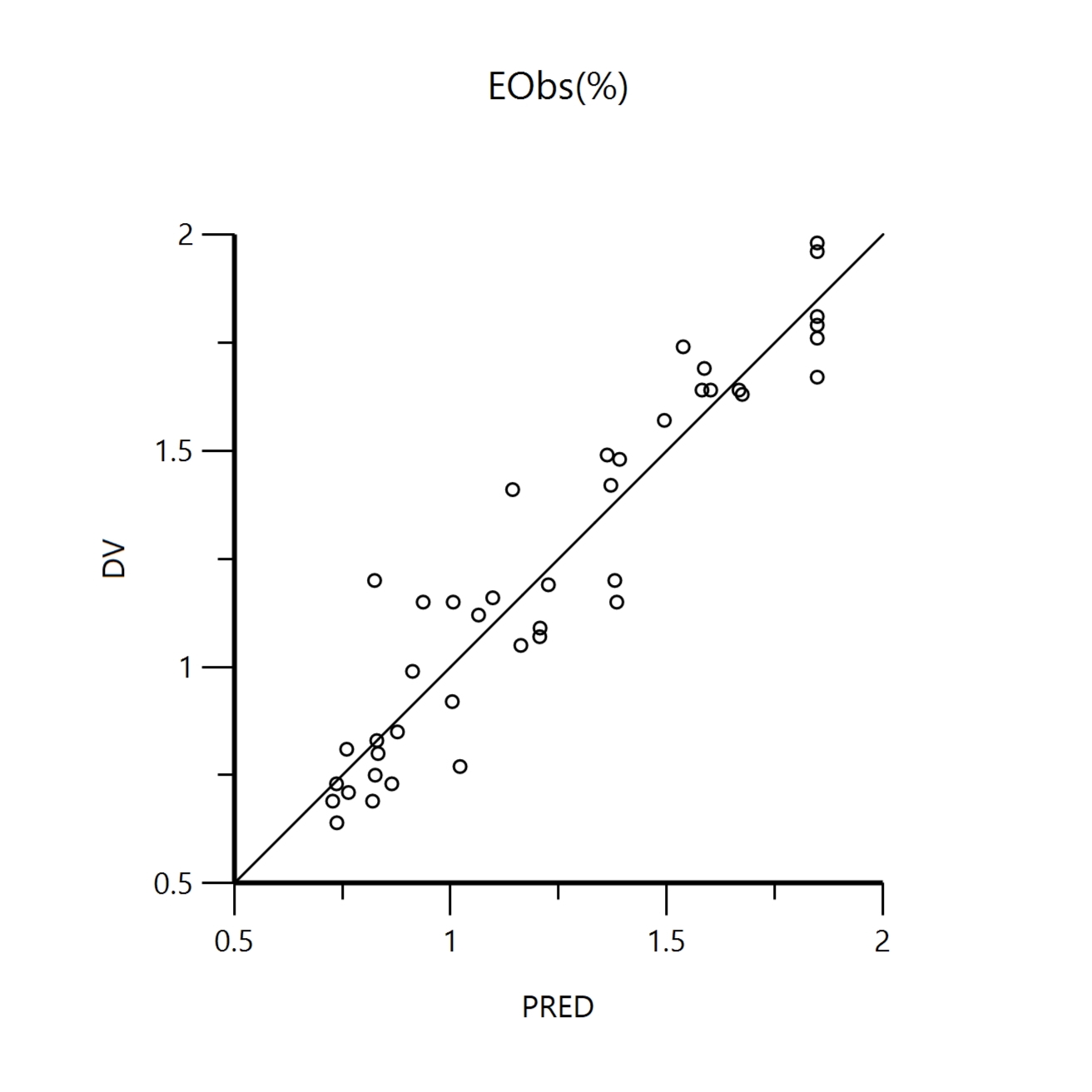

Supplement: Supplementary file 1 [file Image_1.jpeg]

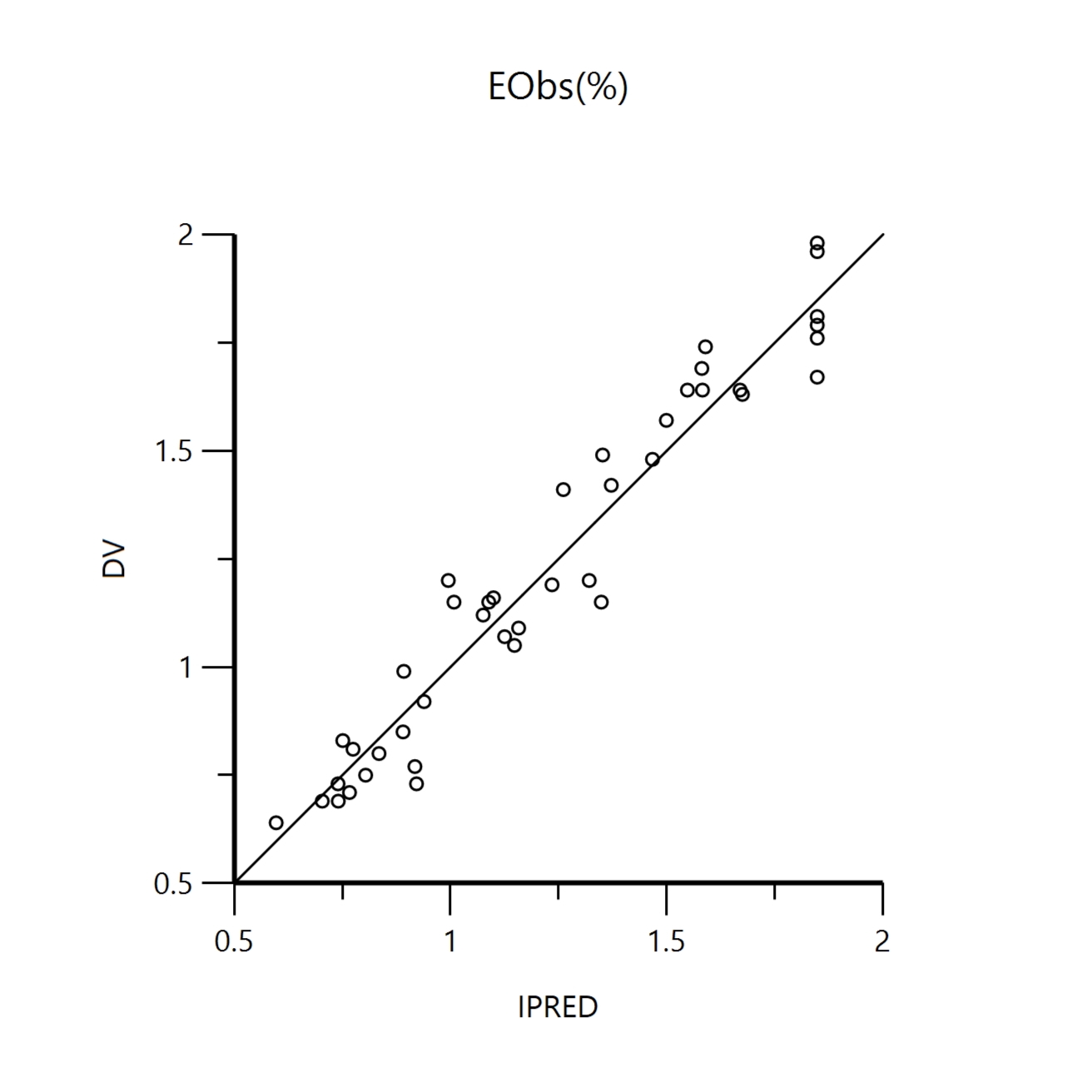

Supplement: Supplementary file 2 [file Image_2.jpeg]
