# Supplementary material for: Increasing plasma ketamine concentrations decrease the minimum alveolar concentration of isoflurane in rabbits
Source: Front Vet Sci. 2025 Jun 19;12:1604553. doi: 10.3389/fvets.2025.1604553 (PMC12221929; doi:10.3389/fvets.2025.1604553)
Supplement: Supplementary file 3 [file Table_3.docx]

Legends for supplemental material

Supplemental 1: Plot of the population predictions vs observations. DV is the dependent variable (observed MAC values) and PRED the population predictions

Supplemental 2: Plot of individual predictions vs observations. DV is the dependent variable (observed MAC values) and IPRED the individual predictions
